# Phylogenetic Relationships of Three Italian Merino-Derived Sheep Breeds Evaluated through a Complete Mitogenome Analysis

**DOI:** 10.1371/journal.pone.0073712

**Published:** 2013-09-09

**Authors:** Hovirag Lancioni, Piera Di Lorenzo, Simone Ceccobelli, Ugo A. Perego, Arianna Miglio, Vincenzo Landi, Maria T. Antognoni, Francesca M. Sarti, Emiliano Lasagna, Alessandro Achilli

**Affiliations:** 1 Dipartimento di Chimica, Biologia e Biotecnologie, Università degli Studi di Perugia, Perugia, Italy; 2 Dipartimento di Biologia Applicata, Università degli Studi di Perugia, Perugia, Italy; 3 Dipartimento di Agronomia Animali Alimenti risorse Naturali e Ambiente, Università degli Studi di Padova, Padova, Italy; 4 Dipartimento di Patologia, Diagnostica e Clinica Veterinaria, Università degli Studi di Perugia, Perugia, Italy; 5 Departamento de Génetica, Universidad de Córdoba, Cordoba, Spain; University of Florence, Italy

## Abstract

In Italy, the crisis of the wool industry triggered the necessity to reconvert the two traditional Merino-derived breeds, Gentile di Puglia and Sopravissana, to meat production, by creating the Merinizzata Italiana. The aim of the present study was to assess the genetic diversity of these three Italian Merino-derived (IMd) breeds by examining the molecular information encoded in the maternally-inherited mitochondrial DNA (mtDNA). A parallel molecular investigation was performed on the putative paternal and maternal breeds, the Merino from Spain and the Appenninica from Italy, respectively, as well as on three unrelated dairy breeds (Sarda and Comisana from Italy, and Lacaune from France). Firstly, the mtDNA control region of 291 samples was analyzed. When comparing the overall genetic distances among the eight stocks, the three IMd breeds clustered together close to the Appenninica, thus confirming its parental role. Among the 90 IMd samples, 82 different haplotypes were observed, almost all belonging to haplogroup B, and only one to A. For 23 mtDNAs, including nine IMd, the analysis was then brought to the level of entire mitogenomes. Three distinct sub-haplogroups within B were found to encompass the IMd samples, with one clade (B1a2a1) apparently restricted to those sheep. Thus, despite experiencing a drastic reduction in number (mainly due to changes in breeding practices driven by the economy), the IMd breeds still represent a reservoir of distinctive mitochondrial variants, which could potentially contribute to the development of conservation and management programs of Italian sheep breeds.

## Introduction

Following domestication in South West Asia approximately 8,000–9,000 years ago, sheep gradually populated a wide geographic range due to their ability to adapt to poor diets and their tolerance to extreme climatic conditions. However, quite recently, some local breeds have been lost by substitution or crossbreeding with commercial breeds. The loss of diversity in livestock species has important economic, ecological, and scientific implications, as well as social considerations. In Italy, sheep breeding has always played an important role, mainly in less-developed and rural areas, where local traditions and native breeds strongly influenced the production systems [1]. For about half a millennium, the two traditional Italian Merino-derived (IMd) breeds, Gentile di Puglia and Sopravissana, represented the most important sheep resource in central and southern Italy. The breed Gentile di Puglia (literally “Gentle Apulian”, in reference to its fine wool) originated from repeated introgression (starting in 1435) of original Spanish Merino rams into native ewes from Apulia. The breed Sopravissana (literally “above Vissana”, see below) derived from sporadic introgression (1792) of Merino-Rambouillet rams (from France) into Vissana ewes (from Visso, in the Sibillini Mountains of the Apennines), a now extinct central Italian local breed that could be considered the ancestor of the extant Appenninica (from the Apennines) sheep [2], [3]. In the last few decades, the increased demand for meat, the gradual abandonment of transhumance practices, and the advent of artificial fibers triggered the necessity to convert the IMd breeds, historically tied to transhumance in central-southern Apennines, to meat production. In many flocks, these sheep were used to create a large crossbred population that eventually resulted in the most popular Italian sheep for meat, officially established in 1989 and named Merinizzata Italiana [4]–[6]. An undesired consequence was the strong numerical reduction of the two local breeds (e.g. Sopravissana decreased from >1 million to a few thousand), which eventually became endangered, according to the European Council Regulation (EEC) No. 2078/92. Currently, a major objective is the preservation of the residual genetic variability of the two threatened Merino-derived breeds (Sopravissana and Gentile di Puglia) and to avoid the improper registration of some Merinizzata Italiana animals within the two native breeds because of their similar morphology [6].

Molecular genetics has proven highly informative to better understand the (recent) evolutionary history of livestock breeds, as well as to determine the level of their genetic variability, which is an essential aspect to consider when defining conservation priorities and regional breed-specific programs [7]–[17]. The reported history of the three IMd breeds was recently confirmed by comparing their microsatellite profiles (based on 30 autosomal markers) with those of the Spanish Merino [18]. Here, we present additional information on the genetic history of these breeds obtained from the analysis of the maternally-inherited mitochondrial DNA (mtDNA). While the informative power of the mitogenome has been widely used to investigate human evolution [19], it has also brought new insights to the analysis of domesticated species [20]–[23]. Until now, molecular surveys of ovine mtDNA have been mostly restricted to the short hypervariable region, with the reported haplotypes resulting in the identification of a few maternal lineages, which are poorly correlated to specific geographical distributions [11], [15], [21], [24]–[47]. The aim of the present study was to assess the matrilineal genetic diversity of the Italian Merino-derived breeds and to further investigate their origins through a refined analysis of their entire mitogenomes. The phylogenetic reconstruction was also enriched through a parallel analysis of two closely related breeds (Spanish Merino and Appenninica), and three unrelated dairy breeds (Lacaune, the most widely used dairy sheep in France; Comisana, a breed from Sicily mainly raised for milk; and Sarda, a Sardinian sheep primarily used to produce milk for distinctive cheeses, such as Pecorino Sardo).

## Results

**Figure 1:**
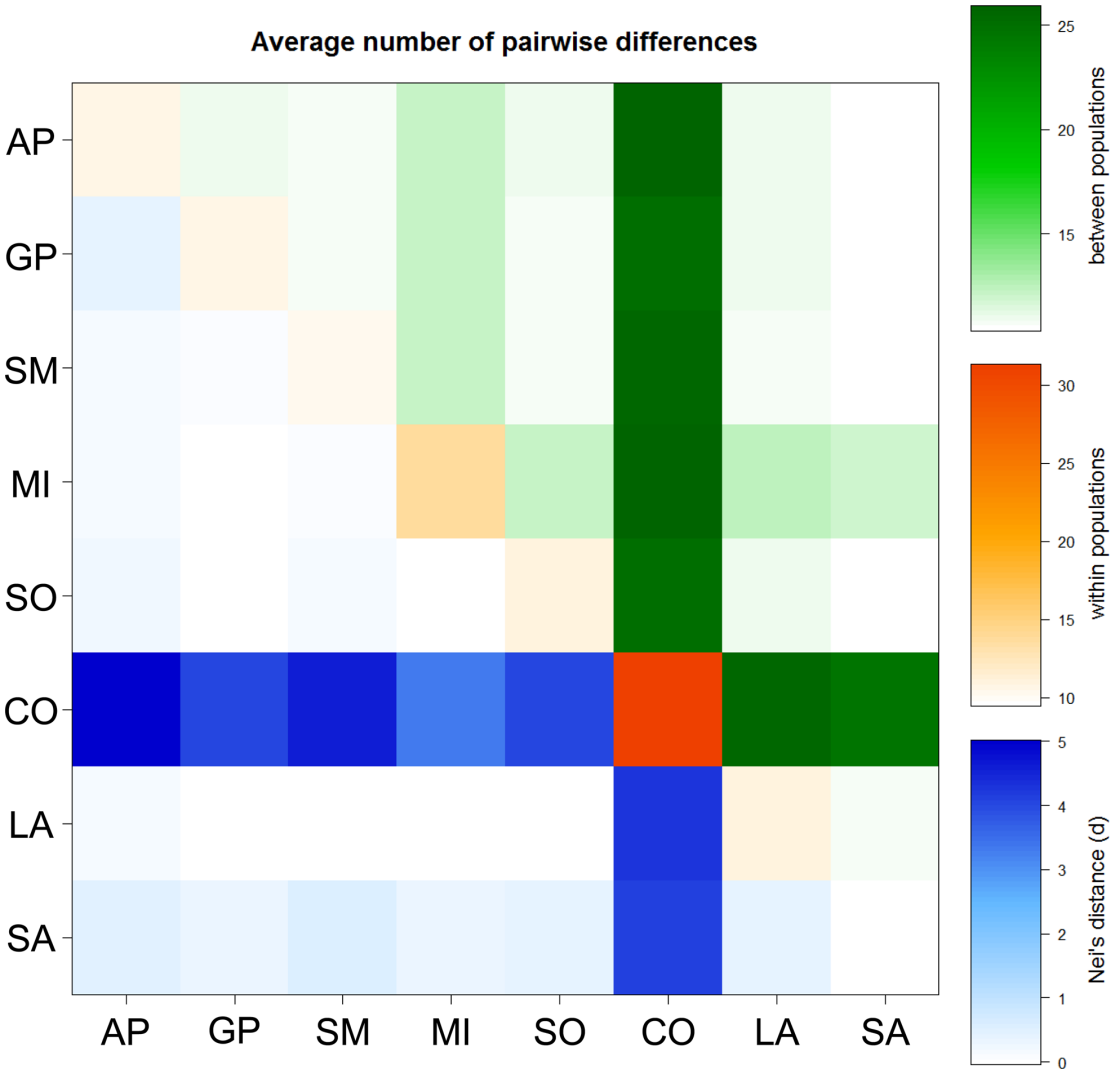
Plot of pairwise population genetic distances. Data were obtained by the concomitant analysis of all breeds subdivided into two groups: (1) SM, AP, SO, GP, MI; (2) SA, CO, LA.

**Figure 2:**
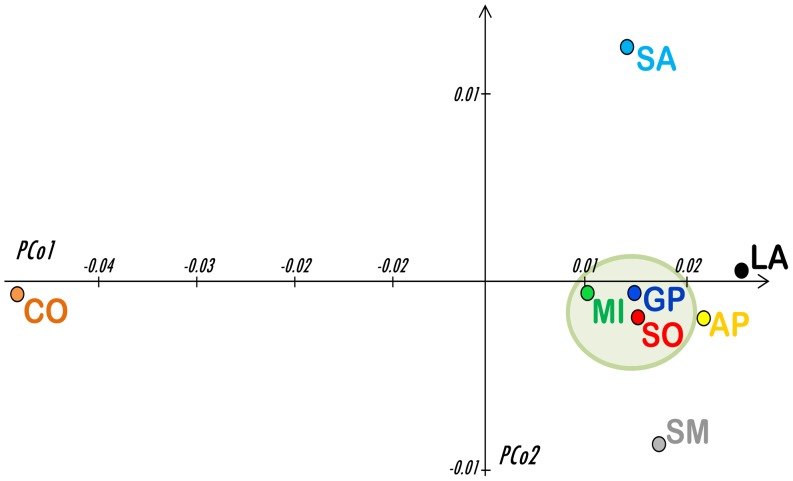
PCo analysis. The plot is based on pairwise genetic distances among the eight breeds (indicated by different colors) and based on 291 control-region haplotypes. The IMd cluster is indicated by the oval.

**Figure 3:**
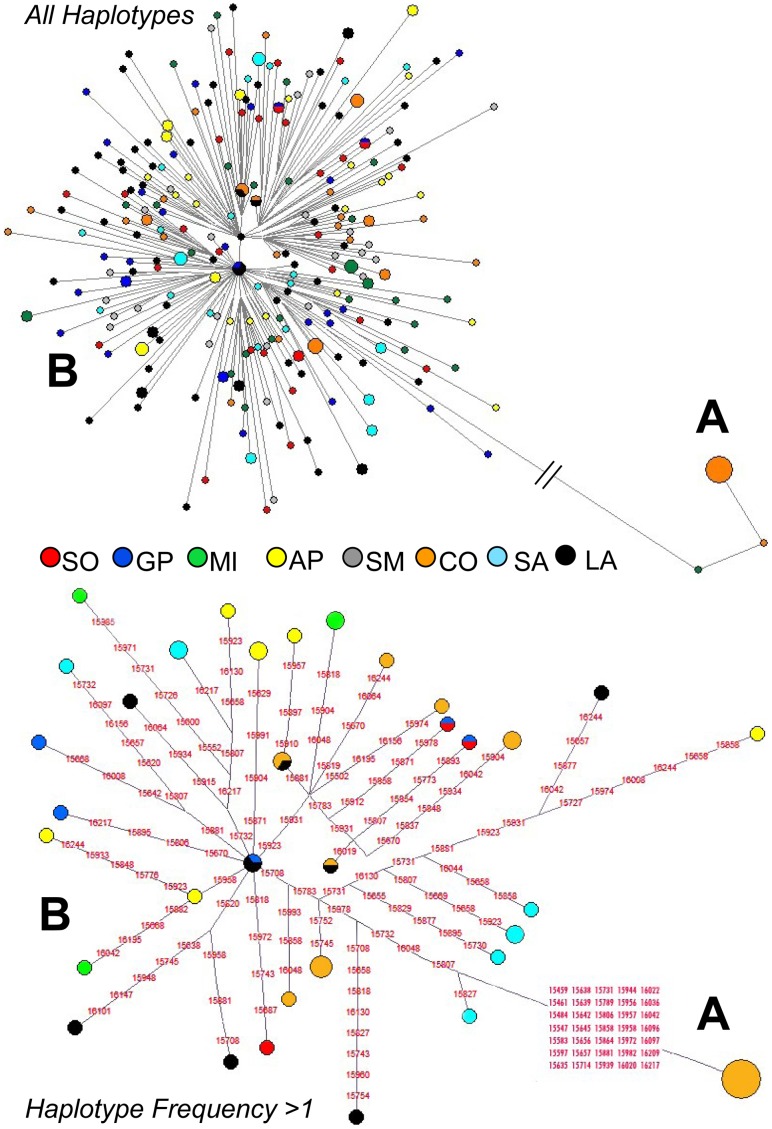
Median-joining networks of the sheep control-region sequences. The top network was obtained from the entire dataset, while the bottom one was acquired by activating the haplotype frequency>1 option.

A total of 812 base pairs (bps) of the mitochondrial control region (from np 15,452 to np 16,263) were obtained for all the 291 samples (Table S1). Excluding ambiguous sites, the overall sequence alignment revealed 221 polymorphic sites (*S*), all represented by single nuclear polymorphisms (SNPs), with an overall nucleotide diversity (π) of 0.016 (Table 1). The average number of nucleotide differences (κ) between two randomly chosen sequences was 12.6. A total of 80 singletons (mutations from the reference sequence appearing only in a single animal) and 235 different haplotypes (nh) were identified, with an observed haplotype diversity (Hd) higher than 0.99 across all breeds (Table 1). When comparing haplotype and nucleotide diversity indices (calculated within each breed), no significant differences were observed. The highest haplotype diversity was detected in the Spanish Merino (Hd = 1.0), where all observed sequences were different, and the lowest in the Comisana (Hd = 0.920). However, the fewer Comisana haplotypes are characterized by a higher number of nucleotide variants, as indicated by their greatest variability in the nucleotide diversity (π = 0.028) (Table 1). As shown hereafter, the reason for this peculiarity becomes evident when analyzing the phylogenetic relationships of the control-region haplotypes. Using an analysis of molecular variance (AMOVA), we examined fixation indices in two (artificially created) population groups, one including the Merino related breeds (SM, AP, SO, GP, MI) and the other encompassing the remaining control-stocks (SA, CO, LA). The among-breeds component of genetic variation is higher for the control-stocks than for the “Merino-related” breeds (by ∼14%). In fact, the vast majority of the observed variance (98.93%) within those five breeds is attributable to differences among samples within breeds, with only 1.07% of differences represented among breeds (Table S2). The overall AMOVA analysis, which concomitantly incorporates a hierarchical grouping of populations, revealed no significant differentiation among the two groups (0.15%, Φ_CT_ = 0.0015, P = 0.34) and confirmed that the source of variation can be mostly (and significantly) attributed to differences among samples within breeds (91.08%, Φ_ST_ = 0.089, P<0.01). According to the pairwise, population-by-population, genetic distances (Figure 1), Lacaune is closely related to all other breeds, whereas Comisana is the most genetically distant and it is also the most heterogeneous breed. A Principal Coordinate (PCo) analysis was also performed to better visualize the breed genetic relationships. Figure 2 illustrates the two PCos. In the third quadrant, where the Spanish Merino (SM) falls, the three IMd breeds (GP, SO and MI) and the Appenninica (AP) form a cluster of closely related breeds. We also observed that the first coordinate (PCo1) clearly separates the Comisana from the other breeds. The genetic differentiation of this breed is confirmed (and explained) by the network analysis of the control-region haplotypes (Figure 3, top). Most samples fall into a single clade with sub-branches radiating from a central node in a typical star-like fashion. The only notable exception is represented by few mtDNAs that cluster into a distinct branch separated by 35 mutations. This second cluster includes one Merinizzata Italiana and about a third of the Comisana samples (13 out of 45). The two distinct clades correspond to lineage affiliations that were previously reported as haplogroups B and A, respectively [24], [35]. Of particular note is the network obtained by activating the frequency >1 option (Figure 3, bottom). This clearly shows that hardly any haplotype is shared among different breeds. The only two haplotypes found to be identical across the IMd breeds are observed in Sopravissana and Gentile di Puglia. Two additional haplotypes are shared between the Comisana and Lacaune breeds, and one more is shared between Gentile di Puglia and Lacaune. In this analysis, haplogroup A is represented by a single haplotype identical in 12 Comisana samples, while haplogroup B includes, among others, 28 shared haplotypes distributed among different breeds. As expected by the Hd value, the Spanish Merino are not represented in this network because their mtDNAs are all different.

**Table 1:** Estimates of genetic diversities a and haplogroup frequencies b for each sheep breed.

Breed	ID	Origin	N^a^	π	nh	Hd	*S*	HgA^b^	HgB^b^
**Sopravissana**	SO	ITALY, CENTRE	30	0.012	29	0.998	80	0 (0.00)	30 (1.00)
**Gentile di Puglia**	GP	ITALY, SOUTH	30	0.012	28	0.995	69	0 (0.00)	30 (1.00)
**Merinizzata Italiana**	MI	ITALY, CENTRE-SOUTH	30	0.015	27	0.991	95	1 (0.03)	29 (0.97)
**Appenninica**	AP	ITALY, CENTRE	30	0.012	25	0.989	71	0 (0.00)	30 (1.00)
**Spanish Merino**	SM	SPAIN	26	0.012	26	1.000	70	0 (0.00)	26 (1.00)
**Comisana**	CO	ITALY, SICILY	45	0.028	25	0.920	85	13 (0.29)	32 (0.71)
**Sarda**	SA	ITALY, SARDINIA	30	0.011	22	0.977	56	0 (0.00)	30 (1.00)
**Lacaune**	LA	FRANCE, ROQUEFORT	70	0.012	63	0.997	112	0 (0.00)	70 (1.00)
**TOTAL**			291	0.016	235	0.997	221	14 (0.05)	277 (0.95)

a N = number of analyzed samples; π = nucleotide diversity; nh = number of unique haplotypes; Hd = haplotype diversity; *S* = number of polymorphic sites.

b Relative frequencies are in parentheses.

**Figure 4:**
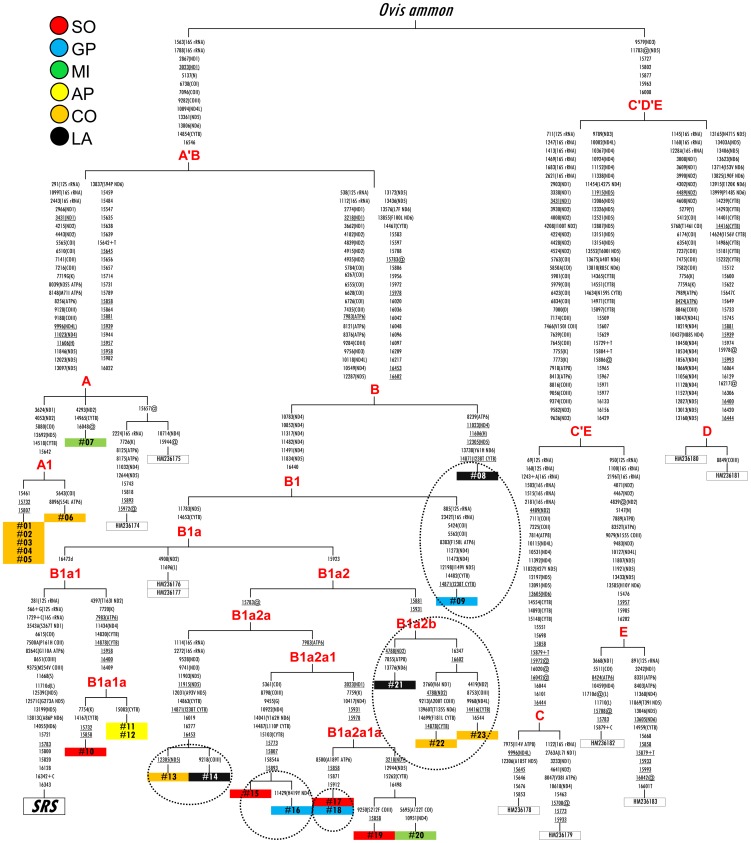
A parsimonious tree of complete mtDNA sequences from sheep. This tree was rooted by using the published argali (*O. ammon*) sequence (GenBank: HM236188). Mutations are shown on the branches and are numbered according to the sheep reference sequence (SRS, GenBank: NC_001941.1). Mutations are transitions unless a base is explicitly indicated. Suffixes indicate transversions (to A, G, C, or T), while “+ and d” denote insertions and deletions, respectively. Recurrent mutations are underlined. The samples are highlighted with different colors to identify their breed of origin. Control-region shared haplotypes (from np 15,452 to np 16,263; Figure 3) are indicated by dotted ovals. Additional information regarding each mtDNA is available in Table 2.

On the basis of these control-region data, we selected for complete sequencing those samples with shared mtDNAs as well as the most divergent ones (with the aim of including the largest possible range of mtDNA variation). The 23 complete mitogenomes were compared with the sheep reference sequence (SRS, GenBank: NC_001941) along with 10 published records (GenBank: HM236174 - HM236183) (Table 2). Figure S2 illustrates the variation of nucleotide diversity (π) along the entire mitogenome. As expected [21], the highest diversity was observed around the control region (from np 15,437 to np 16,616), with a peak of ∼0.04. The overall evolutionary history of the 33 mitogenomes was inferred by a parsimony approach, using the published argali (*Ovis ammon*) sequence (GenBank: HM236188) as an outgroup (Figure 4). As for samples sharing the same control-region haplotype (between nps 15,452 and 16,263), only one set (composed of Gentile di Puglia and Sopravissana) was found to be identical at the whole molecule level, while a single mutation (at np 11429) separates the Gentile di Puglia from the Sopravissana samples, and another set of mutations (at nps 9218 and 12305) distinguishes the Comisana from the Lacaune subjects. Lastly, several mutations separate the remaining samples that shared control-region haplotypes.

**Table 2:** Sources and haplogroup affiliation of the sheep complete mtDNA sequences.

	*ID#* a	*Original name*	*Haplogroup*	*Breed*	*GenBank*
	Outgroup	*Ovis ammon*			HM236188
Novelmitogenomes	01	CO014	A1	Comisana	KF302440
	02	CO015	A1	Comisana	KF302441
	03	CO020	A1	Comisana	KF302442
	04	CO021	A1	Comisana	KF302443
	05	CO026	A1	Comisana	KF302444
	06	CO017	A1	Comisana	KF302445
	07	MI066	A	Merinizzata Italiana	KF302446
	08	LA021	B	Lacaune	KF302447
	09	GP117	B1	Gentile di Puglia	KF302448
	10	SO006	B1a1a	Sopravissana	KF302449
	11	AP038	B1a1a	Appenninica	KF302450
	12	AP053	B1a1a	Appenninica	KF302451
	13	CO002	B1a2a	Comisana	KF302452
	14	LA068	B1a2a	Lacaune	KF302453
	15	SO012	B1a2a1	Sopravissana	KF302454
	16	GP092	B1a2a1	Gentile di Puglia	KF302455
	17	SO030	B1a2a1a	Sopravissana	KF302456
	18	GP105	B1a2a1a	Gentile di Puglia	KF302457
	19	SO009	B1a2a1a	Sopravissana	KF302458
	20	MI075	B1a2a1a	Merinizzata Italiana	KF302459
	21	LA047	B1a2b	Lacaune	KF302460
	22	CO027	B1a2b	Comisana	KF302461
	23	CO046	B1a2b	Comisana	KF302462
Publishedmitogenomes	HM236174	HM236174	A	Merino	HM236174
	HM236175	HM236175	A	Romney	HM236175
	HM236176	HM236176	B1a	Karakas	HM236176
	HM236177	HM236177	B1a	Karakas	HM236177
	HM236178	HM236178	C	Karakas	HM236178
	HM236179	HM236179	C	Morkaraman	HM236179
	HM236182	HM236182	E	Awassi	HM236182
	HM236183	HM236183	E	Tuj	HM236183
	HM236180	HM236180	D	Morkaraman	HM236180
	HM236181	HM236181	D	Morkaraman	HM236181
	SRS	NC_001941	B1a1	Merinolandschaf	NC_001941

a Labeled as in Figure 4.

The tree topology reveals two major clusters (here named A’B and C’D’E) that radiate early in the tree and include all the five haplogroups known to date. Our samples are confirmed as belonging to the A and B branches, but spread across different derived sub-groups. Haplogroup A includes one Merinizzata Italiana and shows a major (most frequently represented) sub-clade, here named A1, encompassing six Comisana samples. Haplogroup A was first thought to be confined to animals with an Asiatic origin, [47], but it was later identified, although at a much lower frequency, in European breeds as well [15], [21], [34], such as the British Romney (HM236175), and breeds from the south western fringe of Europe including the Spanish Merino (HM236174). Haplogroup B, which is considered a typical European lineage, exhibits a more complex sub-structure with two early splits (one marked by a Gentile di Puglia) that predate a major sub-clade named B1a. Excluding two previously published Karakas ewes from Turkey, the B1a mitogenomes cluster into two sub-groups. B1a1 contains the SRS and three of our samples (grouped as B1a1a): two Appenninica and one Sopravissana. B1a2, which also includes various breeds, could be further subdivided into B1a2a and B1a2b. It is worth noting that a coding transition at np 7983 discriminates a clade (B1a2a1) made only of IMd mtDNAs. The remaining six published mtDNAs also allowed us to propose a preliminary full-mitogenome phylogeny of the three remaining sheep haplogroups (C, D and E) identified thus far. Even if the complete mitogenome of each haplogroup was previously published [21], through the updated phylogeny reported in this study, it is possible to define all unique clades by a specific motif (relative to SRS) encompassing both the coding and the control regions (Table S3).

## Discussion

Currently, there are just a few genetic studies aiming to improve the knowledge of the genetic composition of Italian sheep breeds and often they are limited to the analyses of the nuclear genome [18], [48]–[51]. Mitochondrial data for Italian breeds were previously reported only by Pariset and colleagues [45] that performed phylogenetic analyses on mtDNA control-region haplotypes and nuclear polymorphisms of Gentile di Puglia and four other Italian sheep breeds. The aim of the present study was to assess the diversity of IMd breeds and to obtain more information on their maternal origins. A comparative mtDNA analysis included also two closely related breeds (Spanish Merino and Appenninica) and a control-group of two (insular) Italian and one French breeds (Comisana, Sarda and Lacaune, respectively). The mtDNA highest variable segment was preliminarily analyzed in order to identify molecular similarities and phylogenetic relationships. Genetic diversity considered in terms of number of haplotypes and nucleotide diversities showed the same high level as presented in previous ovine mtDNA studies [11], [24], [30], [31]. When comparing the overall genetic distance of the eight analyzed stocks, the three IMd breeds form a closely related cluster near to their female ancestor breed (Appenninica), which might reflect recent maternal gene flow among them. Once distributed throughout a median-joining network, the numerous haplotypes resulted in two haplogroups (A and B), which are consistent with previous studies on domestic sheep breeds in Italy [45] and in Western Eurasia [15], [24], [30], [31], [35], [45].

The predominant haplogroup B (95% in total; 99% in IMd breeds) shows a star-like pattern typical of livestock species and interpreted as recent population expansions (e.g. following domestication) [52]. Haplogroup A (5% in total; 1% in IMd breeds), herein identified mostly in Comisana and in one sample (#07) of the Merinizzata Italiana breed, has been previously found in a Merino sample and in (few) other Iberian breeds. While the mtDNA control region initially contributed predominantly to the description of sheep genetic diversity (Figure S2), the detection of several additional variants was made possible through the molecular analysis of the entire mitochondrial genome. Thus, the IMd mitogenomes were assigned to different sub-clades, one of them (B1a2a1) comprising only IMd samples. Moreover, the identical nature of just one of the five shared control-region haplotypes (shown by samples reported as #17 and #18, Figure 4) was observed on the entire mtDNA, while the remaining cases revealed at least one coding region mutation that discriminates between two samples. Thus, only at the maximum possible level of resolution can the extent of mtDNA information be fully exploited, identifying unexpected genetic variants that would otherwise go undetected.

## Conclusion

The present study is the first example of a mitochondrial phylogenetic analysis of Italian sheep breeds evaluated at the maximum level of resolution. Based on the overall genetic distances calculated from control-region haplotypes and graphed through the PCo analysis, the IMd breeds appear deeply entangled within each other and genetically closer to the Appenninica (their female ancestors) and to the Spanish Merino, with respect to the control breeds analyzed here. In agreement with the first genetic records [18], our mitochondrial data demonstrate the influence of local Italian breeds (i.e. Appenninica) and support the historical origin of the Italian derived Merino breed from the Spanish populations mentioned in the chronicles. These genetic similarities are also confirmed when looking at the haplogroup affiliation of these breeds that, with the only notable exemption of a unique Merinizzata Italiana (#07 in Table 2), belong to the same (typically European) haplogroup (B). However, when dealing with the time frame of about a half millennium, since the origin of the IMd breeds, our analysis must shift to the entire mitogenome in order to discriminate between different sub-haplogroups or even between single haplotypes. The nine IMd mitogenomes belong to different sub-clades, mostly to B1a2a1 (6 out of 9) and reveal only one haplotype shared among them and/or with the closely related Appenninica and Spanish Merino stocks. Thus, it is likely that, even if the IMd breeds share a common maternal origin, many different haplotypes, belonging to different haplogroups (A and B) and sub-haplogroups (B1*, B1a1a, B1a2a1, B1a2a1a), could account for the present mitochondrial gene pool of these Italian stocks. It is apparent that mtDNA variation reflects only maternal inheritance and does not reveal any male-mediated gene flow [16]. However, the novel mitogenomic data of these IMd sheep, as reported here, represents a first step toward the complete genetic characterization of Italian breeds, as well as the ovine mitochondrial phylogeny.

Even though the first complete mtDNA sequence for sheep has been available since 1998 [41], all of the molecular and evolutionary studies of sheep mitochondrial genome have generally focused on a short fragment of the control-region sequence [30], [35], [45], [53]. Very few authors have reported mtDNA phylogeny data based on larger segments encompassing coding regions [11], [24], [32], [46]. It was not until very recently that Meadows and colleagues published the first sheep phylogeny based on a mitogenome panel [21], revealing that the control region was the mtDNA component, which contributed the greatest amount of support to the basal topology of the sheep phylogeny. However, our mitogenome comparison clearly shows the limitations of previous sheep mtDNA studies that confine analysis to a minor fragment of the control region. This approach could still be justified in cases where it was only desired to distinguish between very divergent haplotypes, i.e. belonging to different haplogroups. However, in cases where it is necessary to discriminate between different sub-haplogroups and/or single haplotypes, complete mitogenome analysis is the method of choice. Such enhanced resolution could eventually discriminate more recent evolutionary events, thus also providing information on whether the deliberate selection during the development of modern breeds (after initial domestication) affected mitochondrial genomes. In the present full-mitogenome study, besides the successful discrimination of the sample pairs (four out of five, 80%) sharing the same control-region mutational motif, we identified a typical IMd sub-clade (B1a2a1) and the novel A1 clade identified only in the Comisana breed.

Here, we also provide for the first time a list of coding- and control-region molecular markers for the previously defined haplogroups, as well as for a number of newly defined sub-haplogroups. This work contributes information that is potentially useful in conservation and management programs where genetic information plays a role of primary importance. Finally, this study shows that when employing entire mtDNA sequences, the phylogenetic resolution of animal mtDNA trees is improved; meaning that, to achieve sustainable management of genetic resources, these local breeds should be valued not only in economic terms but also by taking into account their worth as reservoirs of unique diversity [7], [54].

## Materials and Methods

### Ethics Statement

All experimental procedures were reviewed and approved by the Animal Research Ethics Committee of the University of Perugia.

### Animal Sampling

The IMd samples (30 Gentile di Puglia, 30 Sopravissana and 30 Merinizzata Italiana) were described in a previous paper [18], as well as Appennica (30) and Spanish Merino (26) samples. By using the same procedures of sampling and DNA extraction, we included here three unrelated breeds used as control groups: Sarda (30), Comisana (45), and Lacaune (70) (Figure S1). They are commercial breeds strongly selected for milk production, as opposed to the IMd that are selected for their meat and wool.

All the animals were registered in the Herd Book of the corresponding breed. To ensure that the sampling was representative, the animals were randomly selected from diverse flocks. Additionally, in order to minimize the potential for sampling closely related individuals, we selected Merino-related animals that were unrelated for at least two generations and asked the shepherds about the genealogical relationships of the animals belonging to the control breeds.

### Analysis of mtDNA Control-regions

The complete sheep mitochondrial sequence NC_001941 [41] was used to design the primer set 15346for-157rev (Table S4) for the amplification of the control region from np 15,346 to np 00157. DNA amplifications were performed by following previously reported protocols [18]. PCR products were first purified using the ExoSAP-IT® enzymatic system ExoSAP-IT (USB Corporation, Cleveland, OH, USA) and sent to BMR-Genomics s.r.l. (www.bmr-genomics.com) for standard direct Sanger dideoxy-sequencing (in one direction) triggered by the internal primer 15393for (5′ACTATCAACACCCAAAGCTG3′). Electropherograms were visualized, edited and aligned using the Sequencher™ 5.10 software (www.genecodes.com). New PCR amplifications and sequencing reactions were further performed to resolve sequence ambiguities. All sequences were trimmed to the shortest one. Fragments of 812 base pairs (from np 15,452 to np 16,263) eventually resulted from the standardization of the sequencing results.

Indices such as Haplotype diversity (Hd), nucleotide diversity (π) and average number of nucleotide differences (κ) were estimated with DnaSP 5.10 software [55]. Intra- as well as inter-population comparisons were performed based on the number of pairwise differences between sequences and molecular variance (AMOVA). Calculations were performed using the Arlequin v. 3.5 software package [56]. To minimize biases associated with a higher mutation rate for mtDNA, we calculated genetic distances using a Tamura-Nei distance with high among site rate heterogeneity (γ = 0.22), as in [57]. The PCo analysis on pairwise, population-by-population, genetic distances was performed using Excel software implemented by GenAlEx 6.4 software [58].

The evolutionary relationships were investigated through a median-joining network of control-region haplotypes constructed with the Network 4.6 software (www.fluxus-engineering.com) by using the reduced median algorithm (ρ  = 2), followed by the median-joining algorithm (ε = 0). Nucleotide weighting (ω) was adjusted to reflect the difference in mutational frequency among indels (ω = 30), transversions (ω = 20), and transitions (ω = 10), where the least-common event received the highest value. The maximum parsimony (MP) calculation was used to remove unnecessary median vectors and to avoid reticulations, which could be switched off in the results display.

### Analysis of Entire Mitogenomes

Control-region haplotypes were particularly useful to select mtDNAs for whole genome sequencing, which was performed as previously described for humans [59], cattle [22], and horse [23]. The oligonucleotides used to amplify and sequence sheep mitochondrial genomes are shown in Table S4. The GenBank BLAST tool was used to investigate if these oligonucleotides matched NuMtS (nuclear insertions of mitochondrial sequences) in order to avoid amplification of these regions [60]. The phylogeny including 34 sheep mitogenomes was built following a maximum parsimony approach, as described elsewhere [23], [61], and rooted by using the published argali (*O. ammon*) sequence (GenBank: HM236188).

### Accession Numbers

The GenBank accession numbers for the 291 mtDNA control-region sequences reported in this paper are KF228586-KF228853, while those for the 23 complete mitochondrial genomes are KF302440-KF302462.

## Supporting Information

Figure S1 **Geographical sampling areas of the 291 sheep samples.**
(PDF)

Figure S2 **Nucleotide diversity variation (π) along the entire mitogenome.** A schematic linearized genetic map of the mitogenome is presented on the top.
(PDF)

Table S1 **Control-region haplotypes and haplogroup classification of the 291 sheep mtDNAs.**
(XLSX)

Table S2 **AMOVA results for the two breed groups considered.**
(DOCX)

Table S3 **Diagnostic coding- and control-region mutational motifs of sheep mtDNA haplogroups and sub-haplogroups.**
(DOCX)

Table S4 **Oligonucleotides used to amplify and sequence the entire sheep mitogenome.**
(DOCX)
