# Supplementary figures and images for: Phylogenetic Relationships of Three Italian Merino-Derived Sheep Breeds Evaluated through a Complete Mitogenome Analysis
Source: PLoS One. 2013 Sep 9;8(9):e73712. doi: 10.1371/journal.pone.0073712 (PMC3767607; doi:10.1371/journal.pone.0073712)

**Figure S1. Geographical sampling areas of the 291 sheep samples.**

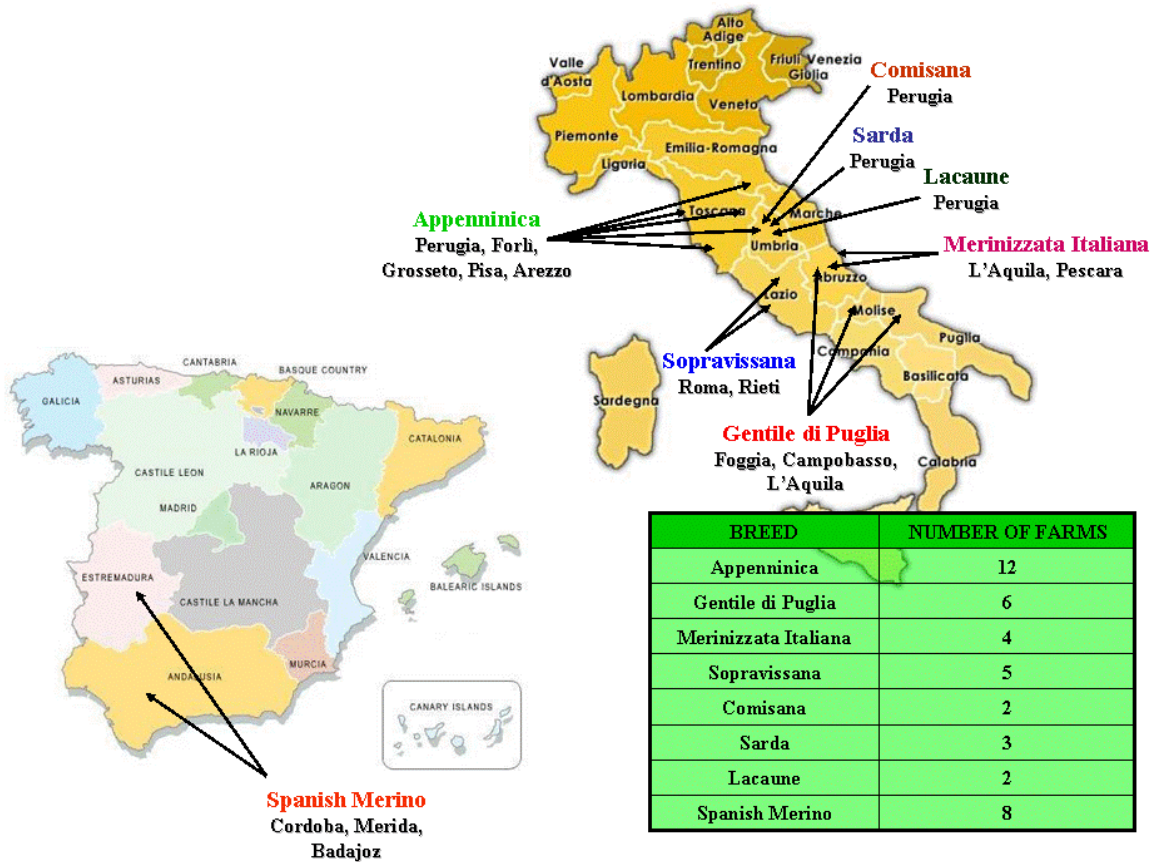

Supplement: Figure S1 — Geographical sampling areas of the 291 sheep samples. (PDF) [file pone.0073712.s001.pdf]

**Figure S2. Nucleotide diversity variation ( $\pi$ ) along the entire mitogenome.**

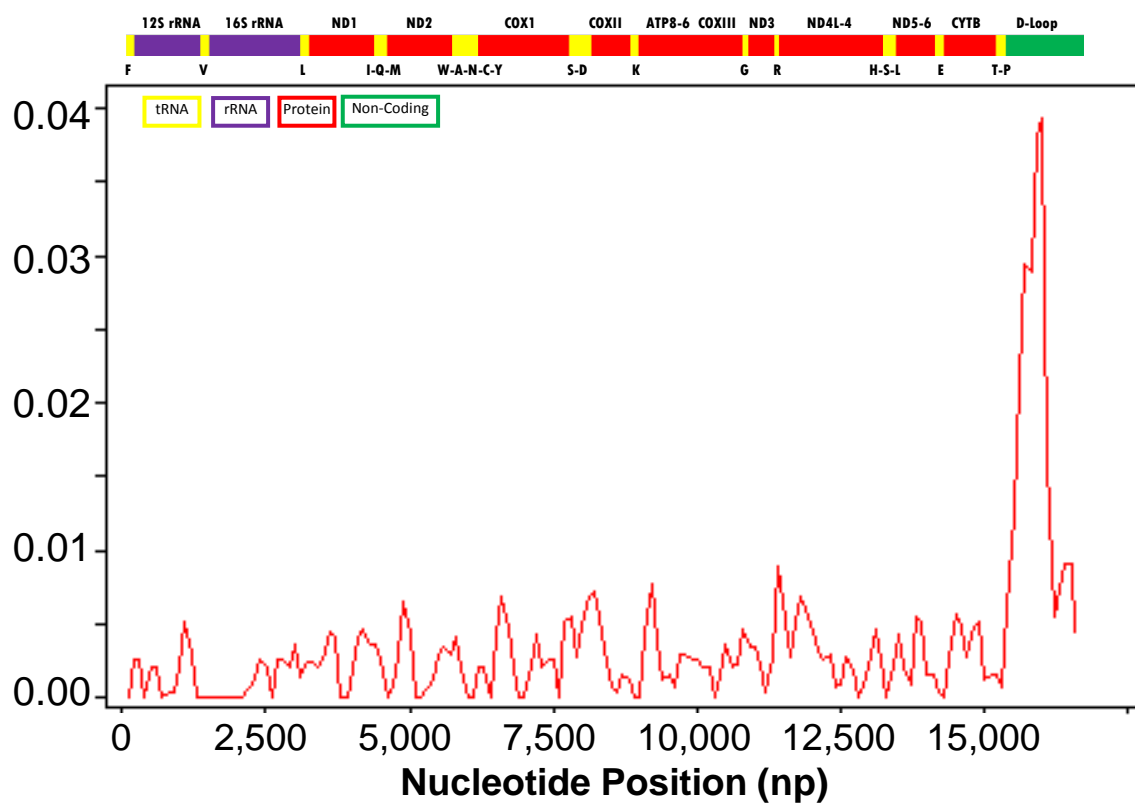

Supplement: Figure S2 — Nucleotide diversity variation (π) along the entire mitogenome. A schematic linearized genetic map of the mitogenome is presented on the top. (PDF) [file pone.0073712.s002.pdf]
