# Supplementary material for: Phylogenetic Relationships of Three Italian Merino-Derived Sheep Breeds Evaluated through a Complete Mitogenome Analysis
Source: PLoS One. 2013 Sep 9;8(9):e73712. doi: 10.1371/journal.pone.0073712 (PMC3767607; doi:10.1371/journal.pone.0073712)
Supplement: Table S2 — AMOVA results for the two breed groups considered. (DOCX) [file pone.0073712.s004.docx]

**Table S2.** AMOVA results for the two breed groups considered.

| Group | Variation  within breeds (%) | Variation  among breeds (%) |
| --- | --- | --- |
| Merino-related breeds | 98.93 | 1.07 |
| Control breeds | 84.96 | 15.04 |

| ΦST (within breeds %) | ΦSC (among breeds, within groups %) | ΦCT (among groups %) |
| --- | --- | --- |
| 0.089 (91.08)  *d.f. 1 - P-value* < 0.01 | 0.088 (8.77)  *d.f. 6 - P-value* < 0.01 | 0.001 (0.15)  *d.f. 283 - P-value* = 0.34 |
