# Supplementary material for: Phylogenetic Relationships of Three Italian Merino-Derived Sheep Breeds Evaluated through a Complete Mitogenome Analysis
Source: PLoS One. 2013 Sep 9;8(9):e73712. doi: 10.1371/journal.pone.0073712 (PMC3767607; doi:10.1371/journal.pone.0073712)
Supplement: Table S3 — Diagnostic coding- and control-region mutational motifs of sheep mtDNA haplogroups and sub-haplogroups. (DOCX) [file pone.0073712.s005.docx]

**Table S3.** Diagnostic coding- and control-region mutational motifs of sheep mtDNA haplogroups and sub-haplogroups.

| **Clade** | **Diagnostic Mutational Motif^a^** |
| --- | --- |
| **A** | 291, 1099T, 2443, 2966, 3431, 4215, 4443, 5565, 6510, 7141, 7216, 7719G, 8039, 8148, 8256, 9128, 9188, 9996, 11023, 11606, 11846, 12023, 13097, 13837, **15459, 15484, 15547, 15635, 15638, 15639, 15642insT, 15645, 15656, 15657, 15714, 15731, 15789, 15858, 15864, 15881, 15939, 15944, 15957, 15958, 15982, 16022** |
| **A1** | 3624, 4053, 5880, 12692, 14518, **15642** |
| **A’B** | 538, 1112, 2774, 3218, 3662, 4182, 4839, 4915, 4935, 5784, 6267, 6555, 6628, 6726, 7435, 7983, 8121, 8376, 9284, 9756, 10118, 10549, 12287, 13172, 13436, 13576, 13855, 14467, **15583, 15597, 15708, 15783@, 15806, 15956, 15972, 15978, 16020, 16036, 16042, 16048, 16096, 16097, 16209, 16217, 16453, 16602** |
| **B** | 10783, 10852, 11317, 11482, 11491, 11834, **16440** |
| **B1** | 11783, 14653 |
| **B1a** | **16472delT** |
| **B1a1** | 281, 566insG, 1729insC, 3543A, 6615, 7500A, 8264C, 8651, 9375, 11668, 11710delC, 12539C, 12571C, 13199, 13813C, 14055, **15721, 15783, 15800, 15820, 16128, 16342insC, 16343** |
| **B1a2** | **15923** |
| **B1a2a** | **15783@** |
| **B1a2b** | **15881, 15931** |
| **B1a2a1** | 7983 |
| **B1a2a1a** | 3023, 7759, 10417, **15931, 15978** |
| **A-E** | 1563, 1788, 2867, 3023, 5137, 6738, 7096, 9282, 10094, 13361, 13806, 14854, **16546** |
| **C’D’E** | 9579, 11783@, **15727, 15802, 15877, 15963, 16008** |
| **C** | 69, 160, 1243insA, 1502, 1515, 2181, 4489, 7111, 7225, 7814, 10115, 10531, 11392, 11832, 12197, 13091, 13605, 14554, 14893, 15148, **15551, 15698, 15858, 15879insT,** [**15972@**](mailto:15972@)**,** [**16020@**](mailto:16020@)**,** [**16042@**](mailto:16042@)**, 16044, 16101, 16444** |
| **D** | 1145, 1160, 1228A, 3008, 3609, 3990, 4302, 4489, 4608, 5279, 5412, 5768, 6174, 6354, 7237, 7475, 7582, 7756, 7759A, 7989, 8424, 8846, 10047, 10219, 10437, 10450, 10534, 10567, 10669, 11056, 11128, 11527, 12827, 13013, 13160, 13165, 13403A, 13406, 13623, 13714, 13825, 13915, 13999, 14239, 14293, 14401, 14416, 14624, 14986, 15181, 15232, **15512, 15600, 15622, 15647C, 15649, 15733, 15745, 15881, 15939, 15974, 15978@, 15993, 16064, 16129, 16217@, 16306, 16400, 16420, 16444** |
| **C’E** | 711, 1247, 1413, 1469, 1683, 2621, 2903, 3338, 3431, 3930, 4008, 4208, 4224, 4428, 4524, 5763, 5850A, 5901, 5979, 6423, 6834, 7000, 7174, 7466, 7639, 7645, 7755, 7773, 7918, 8413, 8816, 9056, 9374, 9582, 9636, 9789, 10002, 10367, 10924, 11152, 11338, 11454, 11915, 12086, 12326, 12521, 12887, 13151, 13154, 13552, 13675, 13810, 14365, 14551, 14634, 14971, 15097, **15509, 15607, 15629, 15729insT, 15804insT, 15806@, 15965, 15967, 15971, 15977, 16133, 16156, 16429** |
| **E** | 950, 1100, 2196T, 4071, 4467, 4839@, 5147, 7889, 8352T, 9079, 9483, 10127, 11807, 11921, 13433, 13585, **15476, 15957, 15985, 16202** |

^a^ Mutational motifs are relative to the reference sequence (SRS, GenBank NC_001941.1), which is a member of haplogroup B. Control-region mutations are in bold.
