# Supplementary material for: Phylogenetic Relationships of Three Italian Merino-Derived Sheep Breeds Evaluated through a Complete Mitogenome Analysis
Source: PLoS One. 2013 Sep 9;8(9):e73712. doi: 10.1371/journal.pone.0073712 (PMC3767607; doi:10.1371/journal.pone.0073712)
Supplement: Table S4 — Oligonucleotides used to amplify and sequence the entire sheep mitogenome. (DOCX) [file pone.0073712.s006.docx]

**Table S4.** Oligonucleotides used to amplify and sequence the entire sheep mitogenome.

| PCR ID^a^ | Fragment  (bps) | Name^b^ | Sequence (5’→3’) | Melting T.  (°C) |
| --- | --- | --- | --- | --- |
| 1 | 1.764 | 21for | AAGCAAGGCACTGAAAATGC | 60.4 |
|  | 1784rev | TGACTTAAACTTGTGCGAGGA | 58.6 |  |
| 2 | 1.843 | 1442for | TGGTGATAGCTGGTTGTCCA | 60.1 |
|  | 3284rev | GGGTCATGCTGGGAAGATTA | 59.9 |  |
| 3 | 1.723 | 2954for | ATTCATTCTAGCCCCCATCC | 60.1 |
|  | 4676rev | GGGGGAAGTCCTCCTATTGA | 60.3 |  |
| 4 | 1.746 | 4458for | AATTGCCCACATAGGCTGAA | 60.5 |
|  | 6203rev | TGGTGGGCTCATACAATGAA | 59.9 |  |
| 5 | 1.735 | 5959for | CTAACGGACCGAAACCTGAA | 60.1 |
|  | 7693rev | TGATGCGGATCATTTTTCAA | 60.0 |  |
| 6 | 1.749 | 7485for | CCGAAGATGTCCTACCCTCA | 60.1 |
|  | 9233rev | GAGGCCGTGAAATCCTGTAG | 59.7 |  |
| 7 | 1.81 | 9018for | TCTGTCCTTCTAGCCTCAGGA | 59.2 |
|  | 10827rev | TTGGGAAGTCAGAGGTGGAG | 60.2 |  |
| 8 | 1.749 | 10642for | ATCCCTGCCCCTATTAGTCG | 60.3 |
|  | 12390rev | GGGGTAGGTTTGAGTCGTTTG | 60.8 |  |
| 9 | 1.795 | 12179for | TGGCTGAGAAGGAGTTGGAA | 60.9 |
|  | 13973rev | GGGTTGGGTGTGGTATTGTG | 60.9 |  |
| 10 | 1.836 | 13706for | ATAACCCAATCCCCCATACC | 59.6 |
|  | 15541rev | GAGTGGGAAGTCCGTGTTGT | 60.0 |  |
| 11^c^ | 1.427 | 15346for | GGAGAACAACCAACCTCCCTA | 60.3 |
|  | 157rev | TGATTCGAAGGGCGTTACTC | 60.2 |  |
|  | | | | |
| Internal Sequencing Oligonucleotides | | | | |
| 1 |  | 594for | CGGTGCTTTATACCCTTCT | 54.5 |
| 2 |  | 2078for | CACGAGGGTTTTACTGTCTC | 54.9 |
| 3 |  | 3518for | CTTCACCATCAAATCGCTAC | 55.8 |
| 4 |  | 5051for | TGAATGCAAATCAACCACTT | 56.6 |
| 5 |  | 6551for | ATACATGAGCCAAAATCCAC | 55.0 |
| 6 |  | 8064for | CTCCCTCCAACAGTGAATAC | 54.6 |
| 7 |  | 9611for | GCCTCCCCTTCTCTATAAAAT | 55.8 |
| 8 |  | 11215for | CCTACAAACACTCCTTCCAC | 54.7 |
| 9 |  | 12785for | CTCCATTATCCACAGCCTAA | 55.3 |
| 10 |  | 14328for | CTGACACAACAACAGCATTC | 55.1 |
| 11 |  | 16032for | ATGCGTATCCTGTCCATTAG | 55.2 |

^a^ The annealing temperature for all PCR reactions is 55°C.

^b^ Nucleotide positions correspond to the sheep reference sequence (SRS, GenBank NC_001941.1).

^c^ The same primer set was also used to amplify the control region.
